# Implications of anti‐ganglioside antibodies in isolated dysphagia following COVID‐19 infection: Case series

**DOI:** 10.1002/brb3.3514

**Published:** 2024-05-02

**Authors:** Sejoon Kim, Jisun Bae, Geun‐Young Park, Sun Im

**Affiliations:** ^1^ Department of Rehabilitation Medicine, Seoul St. Mary's Hospital, College of Medicine The Catholic University of Korea Seoul Republic of Korea; ^2^ Department of Rehabilitation Medicine Bucheon St. Mary's Hospital, College of Medicine, The Catholic University of Korea Seoul Republic of Korea

**Keywords:** anti‐ganglioside antibody, COVID‐19, Dysphagia, SARS‐CoV‐2

## Abstract

**Background:**

There have been multiple reports about the occurrence of dysphagia after the contraction of coronavirus disease 2019 (COVID‐19). However, a detailed pathology and epidemiologic relation between COVID‐19 infection and dysphagia have yet to be established. Here, we report three cases of unexplained dysphagia after COVID‐19 diagnosis, with atypical clinical presentations.

**Case report:**

All patients showed severe isolated lower cranial nerve involvement with dysphagia and aspiration, which required full tube feeding but showed no evidence of limb weakness or sensory symptoms. All tested positive for anti‐ganglioside antibody tests, which all commonly (GD1b, GM1, and GQ1b) are known to have terminal NeuNAc(α2‐3)Gal epitope.

**Discussion:**

We report a series of cases featuring severe, isolated dysphagia post‐COVID‐19 infection, concomitant with positive anti‐ganglioside antibodies. One potential etiology is a variant of Guillain–Barré syndrome. Because only isolated dysphagia with sparing of the facial and extraocular muscles was evident in these cases, we explore the association between anti‐ganglioside antibodies specific to NeuNAc(α2‐3)Gal, which has been frequently associated with the development of bulbar dysfunction. Given that NeuNAc(α2‐3)Gal exhibits an affinity for the spike glycoprotein of SARS‐CoV‐2, a cross‐reaction against NeuNAc(α2‐3)Gal may possibly contribute to isolated dysphagia following COVID‐19 infection.

## Graphical abstract

We report particular cases of three patients with unexplained dysphagia after coronavirus disease 2019 (COVID‐19) diagnosis, who all showed linkage to anti‐ganglioside antibodies against the NeuNAc(Î ± 2‐3)Gal as possible causes.

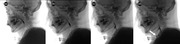

## 1 INTRODUCTION

In addition to affecting respiratory symptoms, coronavirus disease 2019 (COVID‐19) can cause various complications, including cognitive dysfunction, central nervous disorder, peripheral neuropathy, and critical‐illness‐related polyneuropathy (Fiani et al., 2020). Even in the aftermath of recovery, various sequelae can occur, which include lung fibrosis, venous thromboembolism, arterial thromboses, cardiac thrombosis and inflammation, stroke, and overall mood dysfunctions (Desai et al., 2022). There have also been multiple reports about the occurrence of dysphagia after the contraction of COVID‐19. Dysphagia has been broadly associated with poor patient outcomes, including malnutrition, aspiration pneumonia, prolonged hospitalization, and higher mortality (Dawson et al., 2020).

A study with a large sample size reported that nearly 30% of COVID‐19 patients experienced dysphagia, which required therapeutic intervention (Dawson et al., 2020). To date, however, a detailed pathology and epidemiologic relation between COVID‐19 infection and dysphagia have yet to be established. Previous studies have revealed that infection‐related nervous system damage, muscle disease or neuropathy associated with intensive care unit (ICU) stay, and damage to the swallowing structure related to intubation could all serve as possible causes (Kim, 2021). A recent study identified specific risk factors, including ageusia, anosmia, dysphonia, duration of COVID‐19 symptoms, respiratory rate, ICU admission, and the use of noninvasive ventilation, as significant predictors of oropharyngeal dysphagia in COVID‐19 patients (Zayed et al., 2023). By contrast, some may manifest with post‐COVID‐19 infection dysphagia unrelated to the abovementioned causes. We identified anti‐ganglioside antibodies in some patients with unexplained dysphagia that developed after COVID‐19 infection. In this case report, we aim to detail the clinical characteristics of these patients and suggest possible mechanisms that include the possible link of NeuNAc(α2‐3)Gal and its affinity with the spike glycoprotein of SARS‐CoV‐2 as potential mechanisms for post‐COVID‐19 dysphagia. The protocols of this study were approved by the local ethics committee (HC 22ZISI0008). Consent was waived due to the retrospective nature of the study.

## 2 CASE REPORT

### 2.1 Case 1

An 88‐year‐old man was hospitalized in the ICU due to COVID‐19‐related pneumonia. After recovery from COVID‐19, he continued to receive tube feeding and still showed abundant secretions in the airways along with signs of aspiration. After 4 months, he was admitted to the authors’ hospital for dysphagia rehabilitation.

**FIGURE 1:**
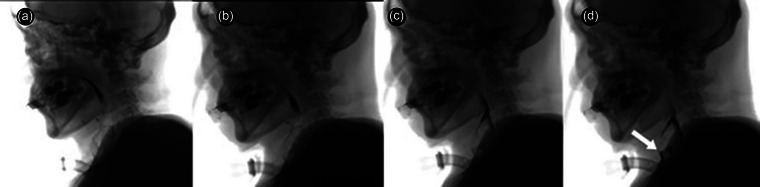
The 0.02‐s interval serial videofluoroscopic swallowing study images of the patient 1, shows decreased epiglottic inversion and poor airway closure which provoke aspiration of barium through the vocal fold (white arrow).

**FIGURE 2:**
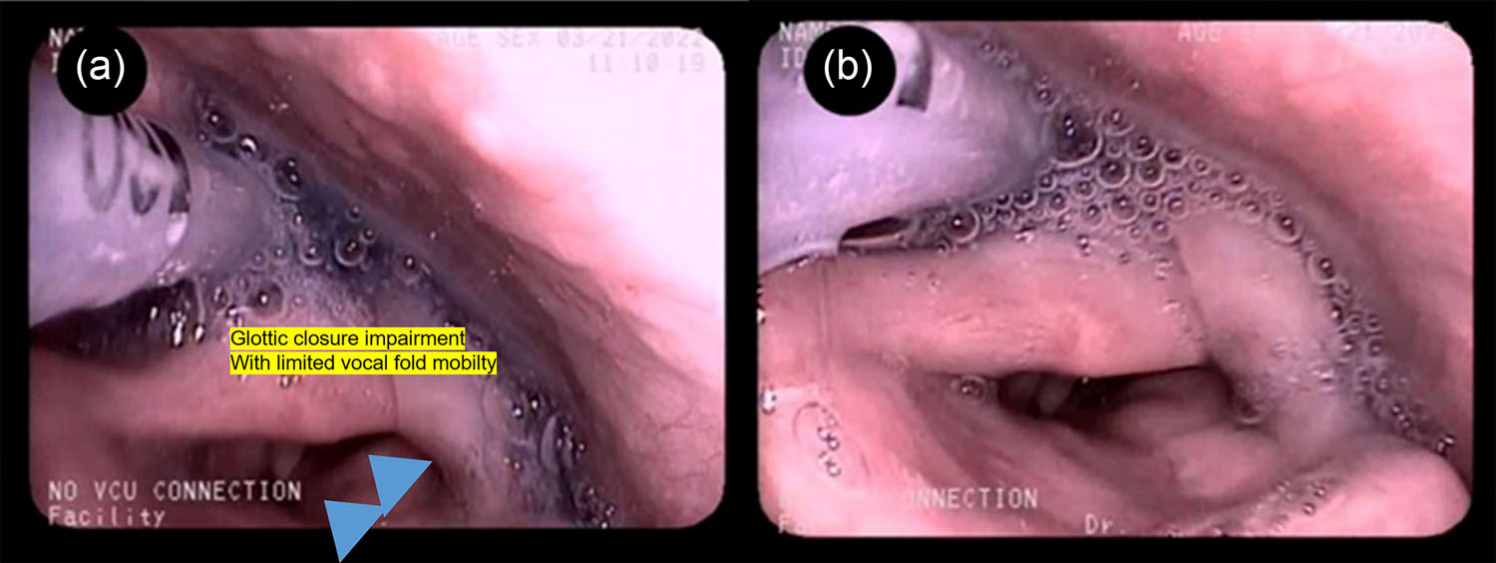
Fiberoptic endoscopy swallowing study of the patient 1 shows (a) residue at pyriform sinus after swallowing attempt of ice with limited vocal fold motion and (b) saliva pooling and overflow aspiration.

The patient had a previous history of diabetes mellitus and paroxysmal atrial fibrillation. He had no prior dysphagia prior to contracting COVID‐19. A videofluoroscopic swallowing study revealed the presence of dysphagia with aspiration (Figure 1), and a fiberoptic endoscopy swallowing study (FEES) showed saliva pooling and aspiration of secretion with vocal fold immobility (Figure 2). On neurological examination, no definite motor or sensory deficit on the extremities was observed, and a baseline laboratory study was within the normal range. The brain's magnetic resonance imaging (MRI) also showed no specific findings. An electrophysiological examination revealed delayed latencies in both the motor and sensory nerves, which is consistent with demyelinating sensorimotor polyneuropathy. Additional serum anti‐ganglioside antibodies testing showed a positive result for anti‐GD1b IgM. The patient showed slight improvement at the follow‐up FEES after 7 months, but he still required tube feeding due to severe dysphagia (Table 1).

**TABLE 1:** Demographics and clinical characteristics of the three cases.

Variables	Patient 1	Patient 2	Patient 3
Age	88	75	72
Sex	Male	Male	Female
History of diabetes	+	–	–
Time interval between dysphagia and COVID‐19	≤7 days	≤7 days	≤14 days
History of post COVID‐19 aspiration pneumonia	+	+	+
History of ICU care	+	–	–
History of MV	+	–	–
Feeding			
Post‐COVID19 dysphagia	Tube feeding	Tube feeding	Tube feeding
Post‐COVID19 6 months	Tube feeding	Tube feeding	Oral feeding
Brain MRI	No acute lesion	No acute lesion	No acute lesion
Electrophysiological examination	Sensorimotor polyneuropathy	Sensorimotor polyneuropathy	Not performed
Anti‐ganglioside antibody	Anti‐GD1b IgM	Anti‐GQ1b IgG	Anti‐GM1 IgM

Abbreviations: COVID‐19, coronavirus disease 2019; ICU, intensive care unit; MRI, magnetic resonance imaging; MV, mechanical ventilation.

### 2.2 Case 2

A 75‐year‐old man was admitted with worsening dysphagia a few days after being diagnosed with COVID‐19. He had a previous history of left middle cerebral artery infarction 8 years ago, but he had been on full oral diet before contracting COVID‐19.

A FEES showed a presence of dysphagia with tongue base weakness with severe impairment in secretion clearance, and he was placed on tube feeding. There was no sensory deficit or sign, facial muscle, or extraocular muscle weakness, with no noticeable decrease of power in the upper and lower limbs. Brain MRI showed no acute lesion. A baseline laboratory study was within the normal range. An electrophysiological examination revealed a demyelinating sensorimotor polyneuropathy. Serum anti‐ganglioside antibodies testing also showed positive results for anti‐GQ1b IgG. Since his dysphagia had persisted despite 4 weeks of rehabilitation therapy, a percutaneous endoscopic gastrostomy was put in place to provide long‐term nutrition (Table 1).

### 2.3 Case 3

A 72‐year‐old woman visited our outpatient clinic complaining of dysphagia. One month before her visit, she had been hospitalized for COVID‐19 but did not receive ICU care due to only having mild respiratory symptoms. Four days after discharge, she was readmitted for aspiration pneumonia and was placed on a nasogastric tube due to the high risk of recurrence of aspiration.

Instrumental swallowing tests also showed severe dysphagia with aspiration, but a baseline laboratory study and brain MRI showed no specific findings. There was no presence of motor and sensory deficits, with no abnormalities of facial or external ocular movement. An electrophysiological examination was not performed due to the patient's refusal. Further studies, including serum anti‐ganglioside antibodies, showed positive results for anti‐GM1 IgM. Her dysphagia improved over time, and after 4 months, she was able to commence a full oral diet (Table 1).

### 2.4 Retrospective case series

**FIGURE 3:**
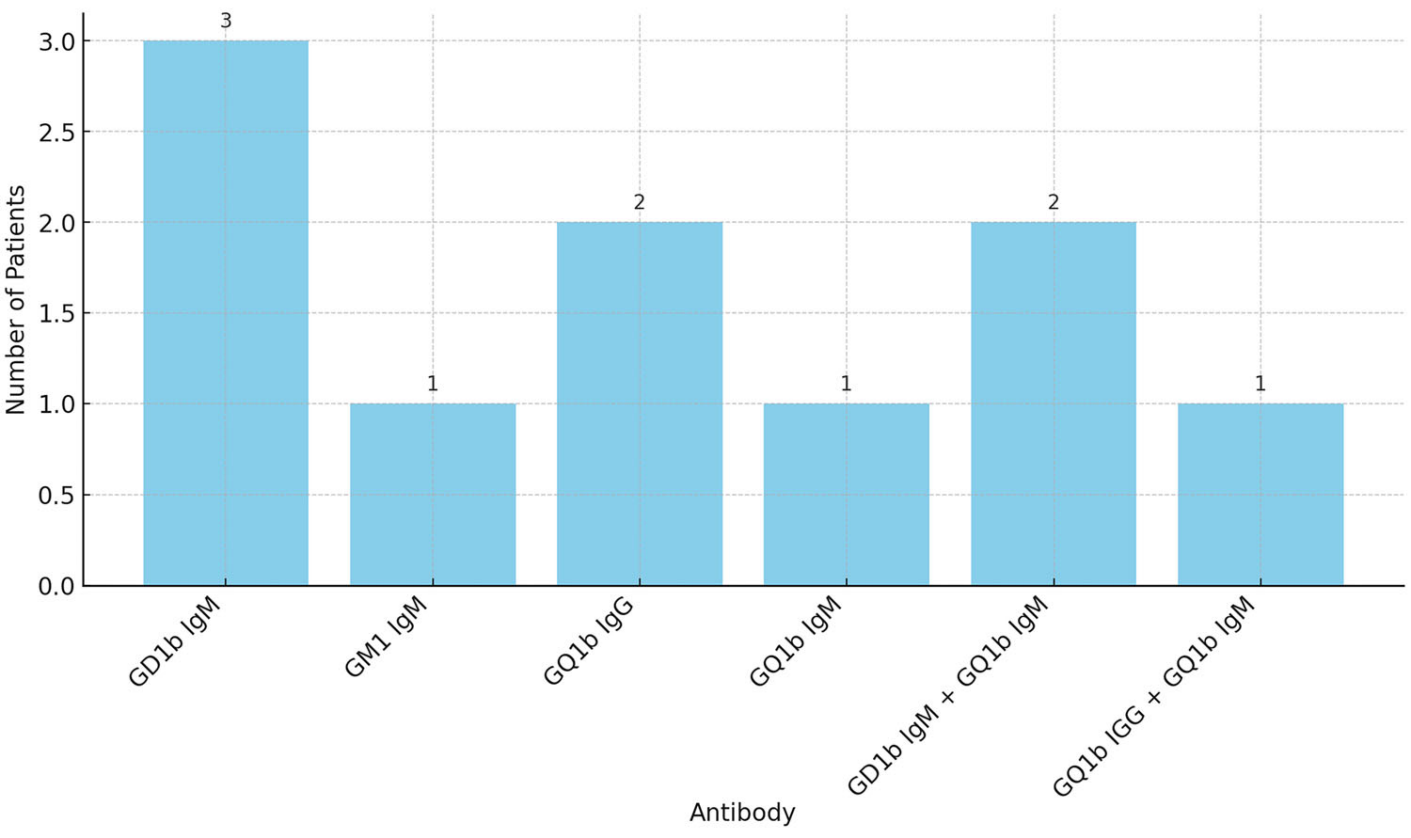
Bar graph depicting the prevalence of anti‐ganglioside antibodies in patients with dysphagia following coronavirus disease 2019 (COVID‐19) infection.

A retrospective analysis of electronic medical records between February 2020 and December 2023 was conducted to identify cases of aspiration pneumonia or dysphagia after COVID‐19 infection. Out of 80 cases referred to our department, 35 demonstrated persistent severe dysphagia exceeding 4 weeks in duration that required total enteral tube feeding, with no other accompanying motor or sensory signs and no etiological factors identified through prior imaging or other diagnostic studies. Including the three cases, a total of 11 patients (31.4%) were seropositive for anti‐ganglioside antibodies (Figure 3). Subsequent exhaustive investigations did not elucidate any alternative significant etiologies for dysphagia in these cases. Consistent with the observed pattern as seen in our three case reports, all these cases showed isolated dysphagia with severe aspiration but no accompanying areflexia, extremity weakness, or ataxia. The majority of these patients necessitated prolonged enteral nutrition via tube feeding, which led to poor prognostic outcomes.

## 3 DISCUSSION

We present three patients who developed dysphagia with aspiration within 2 weeks post‐COVID‐19, necessitating nasogastric tubes and testing positive for anti‐ganglioside antibodies. Despite bulbar muscle dysfunction, no facial or extraocular weakness was observed. Conservative therapies yielded mixed outcomes. An electronic chart review indicated 31.4% of severe post‐COVID dysphagia patients had these antibodies, suggesting a possible link.

Anti‐ganglioside antibodies are frequently detected in the serum of patients with autoimmune neuropathies, which are preceded by viral or bacterial infections. There are several correlations between antibody specificity and clinical symptoms (Emilien & Hugh, 2015; Stathopoulos & Dalakas, 2022). Anti‐ganglioside antibodies have not been previously linked to isolated dysphagia post‐COVID. We propose two theories: atypical Guillain–Barre syndrome (GBS) variants or cross‐reactivity between anti‐gangliosides and the SARS‐CoV‐2 spike protein, given gangliosides’ role in viral cell entry.

The first mechanism relates to dysphagia manifesting as atypical GBS after COVID‐19 (Avenali et al., 2021). GBS is a heterogeneous condition that may be precipitated by various etiological factors, including infectious agents. There is an established association between GBS and the SARS‐CoV‐2 virus. Typically, patients with GBS post‐COVID‐19 infection present with limb weakness and sensory deficits or ataxia. A variant known as Polyneuritis cranialis manifests predominantly with cranial nerve palsies, often without significant limb involvement (Polo et al., 1992). However, our cases do not fulfill the diagnostic criteria for isolated lower cranial nerves—those governing swallowing functions (cranial nerves IX and X), as seen in our cases, with sparing of the facial and extraocular muscles are rare (Han et al., 2017). In fact, this specificity in symptomatology does not align with the established diagnostic criteria for GBS or its variants, which often include ocular and facial muscles involvement.

The second possible mechanism is the cross‐reactivity between the anti‐ganglioside and the SARS‐CoV‐2 spike protein. Gangliosides, composed of a ceramide attached to one or more sugars, contain sialic acid (N‐acetylneuraminic acid, NeuNAc) linked to the oligosaccharide core. They are expressed throughout the body tissues but are particularly strongly expressed within the brain and the nervous system (Cutillo et al., 2020). It has been known that SARS‐CoV‐2 enters human cells by interacting with the angiotensin‐converting enzyme 2 receptor. In addition, recent studies have demonstrated that gangliosides could serve as ligands for the receptor‐binding domain of a SARS‐CoV‐2 spike protein, which facilitates viral entry (Sun, 2021). Although some have commented on the possible neuroinvasive potential of the SARS‐CoV‐2 virus causing the viremia while others have hinted at a secondary immune‐mediated mechanism, the presence of these anti‐ganglioside and the pure bulbar dysfunction manifested in all our cases may indicate this cross‐reactivity of the NeuNAc as one possible mechanism (Khan et al., 2021; Lascano et al., 2020).

The following are some supporting evidence from previous literature that support how anti‐ganglioside antibodies, specifically those targeting NeuNAc(α2‐3)Gal, are implicated in isolated bulbar palsy. Several gangliosides share the terminal NeuNAc(α2‐3) Gal epitope. Oga et al. described IgM or IgG cross‐reactivity against this epitope at first (Oga et al., 1998). However, a specific clinical syndrome linked to this reactivity has not yet been established. Ricard et al. reported that anti‐ganglioside antibodies against NeuNAc(α2‐3)Gal had been frequently associated with the development of bulbar dysfunction presenting dysphagia (Rojas‐Garcia et al., 2010). There were 10 cases of NeuNAc(α2‐3) cross‐reactivity, and five patients among them showed dysphagia. Furthermore, there was a case presenting pure bulbar dysfunction without any other motor or sensory deficits, similar in clinical manifestation to those manifested in this study. Additionally, there has been a case report of a patient who experienced acute bulbar palsy causing dysphagia after an upper respiratory tract infection (Rojas‐Garcia et al., 2006). In that report, similar to this study, the patient had anti‐ganglioside antibodies (GM3, GD1a, and GT1b) that shared the NeuNAc(α2‐3)Gal epitope. Nevertheless, no brain lesions were noted on MRI, nor were there indications of typical GBS as observed in this case series.

All gangliosides in our cases showed positive antibody findings, with identified antibodies being anti‐GD1b IgM, anti‐GM1 IgM, anti‐GQ1b IgM, and anti‐GQ1b IgG—all possessing NeuNAc(α2‐3)Gal. Baker et al. demonstrated that NeuNAc has an affinity with the spike glycoprotein of SARS‐CoV‐2 (Baker et al., 2020). Hence, considering the case report of dysphagia following an upper respiratory tract infection with positive anti‐ganglioside antibodies, this reactivity against NeuNAc(α2‐3)Gal of SARS‐CoV‐2 may elucidate one of the potential mechanisms underlying dysphagia after COVID‐19 infection. This link may explain the distinctive bulbar muscle involvement seen in our cases, without other neurological signs.

Research identifies various potential causes for post‐COVID‐19 dysphagia, including central nervous system damage, ICU‐related muscle disease or neuropathy, and intubation injury. However, the pathogenesis is unclear (Jung et al., 2022; Kim, 2021; Todisco et al., 2021). In our department, 31.4% of patients with unexplained post‐COVID dysphagia had anti‐ganglioside antibodies, suggesting their pathogenic role.

This study adds a novel perspective by demonstrating a significant proportion of post‐COVID‐19 dysphagia cases with anti‐ganglioside antibodies, highlighting a potential new pathogenic mechanism to explore.

Our study has limitations; not all patients had cerebral fluid studies due to their condition and absence of significant motor weakness. Despite ICU discharge, patients continued to experience dysphagia. Treatments like IVIG and corticosteroids are effective for immune neuropathies (Tobon, 2017), but were not used here due to late anti‐ganglioside diagnosis and elapsed symptom onset. At diagnosis, immunotherapy's effectiveness for dysphagia was unsubstantiated. Moreover, anti‐ganglioside's link to post‐COVID conditions and IVIG's benefit for dysphagia were unverified prior to this report, leaving their use in such cases uncertain.

The current research extensively covers acute SARS‐CoV‐2 effects but still lacks insight into long‐term consequences like dysphagia. This report sheds light on the novel link between anti‐ganglioside antibodies and lasting bulbar dysfunction with dysphagia post‐COVID‐19. It offers a new understanding of this rare manifestation and suggests a possible role for anti‐ganglioside antibodies in COVID‐19‐related dysphagia.

## AUTHOR CONTRIBUTIONS

**Sejoon Kim**: Conceptualization; data curation; writing—original draft. **Jisun Bae**: Data curation. **Geun‐Young Park**: Conceptualization; data curation. **Sun Im**: Conceptualization; data curation; writing—review and editing.

## CONFLICT OF INTEREST STATEMENT

The authors declare no conflicts of interest.

### PEER REVIEW

The peer review history for this article is available at https://publons.com/publon/10.1002/brb3.3514.

## Data Availability

The data that support the findings of this study are available on request from the corresponding author. The data are not publicly available due to privacy or ethical restrictions.
